# The Role of KH-Type Splicing Regulatory Protein (KSRP) for Immune Functions and Tumorigenesis

**DOI:** 10.3390/cells11091482

**Published:** 2022-04-28

**Authors:** Kim-Alicia Palzer, Vanessa Bolduan, Rudolf Käfer, Hartmut Kleinert, Matthias Bros, Andrea Pautz

**Affiliations:** 1 Department of Pharmacology, University Medical Center of the Johannes Gutenberg University Mainz, 55131 Mainz, Germany; kpalzer@uni-mainz.de (K.-A.P.); rudi.kaefer@web.de (R.K.); kleinert@uni-mainz.de (H.K.); 2 Department of Dermatology, University Medical Center of the Johannes Gutenberg University Mainz, 55131 Mainz, Germany; vbolduan@students.uni-mainz.de (V.B.); mbros@uni-mainz.de (M.B.)

**Keywords:** KH-type splicing regulatory protein, post-transcriptional gene regulation, mRNA decay, micro RNA, cytokine, antiviral response, T helper cell, tumorigenesis

## Abstract

Post-transcriptional control of gene expression is one important mechanism that enables stringent and rapid modulation of cytokine, chemokines or growth factors expression, all relevant for immune or tumor cell function and communication. The RNA-binding protein KH-type splicing regulatory protein (KSRP) controls the mRNA stability of according genes by initiation of mRNA decay and inhibition of translation, and by enhancing the maturation of microRNAs. Therefore, KSRP plays a pivotal role in immune cell function and tumor progression. In this review, we summarize the current knowledge about KSRP with regard to the regulation of immunologically relevant targets, and the functional role of KSRP on immune responses and tumorigenesis. KSRP is involved in the control of myeloid hematopoiesis. Further, KSRP-mediated mRNA decay of pro-inflammatory factors is necessary to keep immune homeostasis. In case of infection, functional impairment of KSRP is important for the induction of robust immune responses. In this regard, KSRP seems to primarily dampen T helper cell 2 immune responses. In cancer, KSRP has often been associated with tumor growth and metastasis. In summary, aside of initiation of mRNA decay, the KSRP-mediated regulation of microRNA maturation seems to be especially important for its diverse biological functions, which warrants further in-depth examination.

## 1. Introduction

To prevent an exaggerated immune response, tight control of the expression of pro-inflammatory mediators, such as cytokines or chemokines, is necessary. Since these are important regulators of immune cell function, rapid and strong expression is required to elicit a rapid response to an invading pathogen. Nevertheless, it is also important to resolve immune responses to counteract tissue destruction and to prevent autoimmune responses. Gene expression can be controlled by transcriptional and posttranscriptional mechanisms, and the latter comprise regulation of mRNA decay and translation efficiency. The 3-untranslated region (3′-UTR) of mRNA represents an important element in the post-transcriptional regulation of inflammatory cytokines/chemokines by RNA-binding proteins (RPBs) and micro (mi)RNA. In general, RPBs exert rather a stabilizing (human antigen R, HuR) or destabilizing (Tristetraprolin, TTP/KH-type splicing protein, K(H)SRP) effect on mRNA transcript half-life, and may affect its translational efficacy (e.g., T-Cell-Restricted Intracellular Antigen-1) [1,2].

KSRP (K homology [KH]-type splicing protein, KHSRP) is a single-stranded nucleic acid-binding protein which interacts with target RNA species in nuclear and cytoplasmic cell compartments [3]. In humans, the *KSRP* gene is located on chromosome 19p13.3, contains 21 exons, one transcript is listed in the RefSeq database (ENST00000600480.2), and encodes the 747 amino acid (aa) KSRP protein, as investigated in the literature. Some hints from the database indicate that additional transcripts may exist, but nothing is known about their biological significance.

The murine gene (ENSMUSG00000007670) is located on chromosome 17 [4] and contains 19 exons. However, the protein sequence of KSRP (in both species 747 aa) is highly conserved between both species. KSRP was first described in 1996 by Levens laboratory and categorized as a member of the far upstream element (FUSE)-binding protein (FBP) family, and was originally named FBP2 [5]. In addition to KSRP, two other members, named FBP1 and FBP3, belong to that family. FBP1 is involved in different cellular processes by regulating transcription, splicing and translation of target genes [6], whereas the biological function of FBP3 remains largely unknown [7].

**Figure 1:**
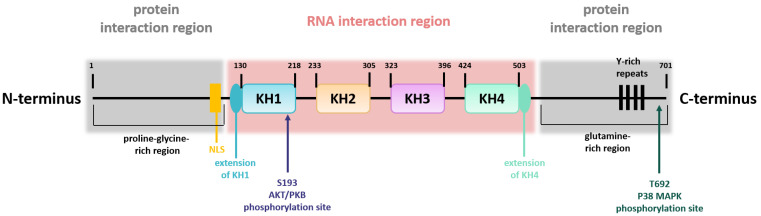
KSRP structure. Schematic overview of KSRP protein structure, including the domain organization in the central region, involved in RNA interaction, and the two N- and C-terminally flanking regions, which are necessary for protein interaction. Phosphorylation sites are indicated (own illustration inspired by [10]).

The structure of the KSRP/FBP2 protein can be divided into a central region that is flanked on either side by one additional region (Figure 1) [8]. The central region contains four KH domains mediating the interaction with single-stranded nucleic acids [9]. Whereas KH domain 1, 2, and 4 bind to a large number of sequence motifs with moderate selectivity, KH domain 3 preferably binds to G-containing sequences. In comparison with the central region, the N- and C-termini are regions with low complexity and are post-translationally modified [8]. The N-terminal part comprises a proline-glycine-rich region, whereas the C-terminal part contains a glutamine-rich region, and both contain elements for protein interaction [5,8]. Moreover, the N-terminal part contains the nuclear localization signal, and the C-terminus harbors four Y-rich repeats. Thus, the structure of KSRP is important for its flexibility in terms of target gene binding. KSRP activity is regulated by phosphorylation and other modifications as outlined below.

**Figure 2:**
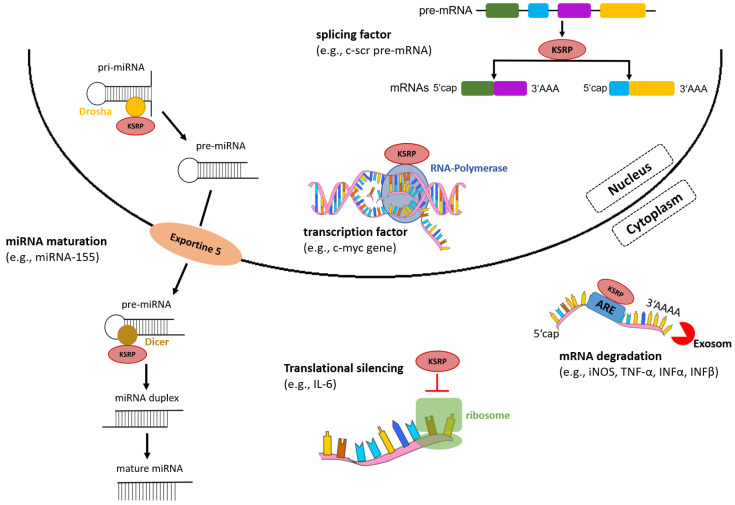
KSRP regulates gene expression on various levels. In the nucleus, KSRP functions as a transcription and splicing factor, and in the cytoplasm mediates rapid decay of ARE-containing mRNAs by recruiting enzymes and silences translation of mRNAs. Moreover, KSRP promotes miRNA maturation by interacting with the ribonucleases Drosha and Dicer (own illustration inspired by [12]).

In the nucleus, KSRP acts as a transcription and splicing factor, and in the cytoplasm regulates mRNA stability by promoting its decay and translational silencing (Figure 2) [11]. In addition to regulation of gene expression via post-transcriptional mechanisms, KSRP promotes the maturation of a subset of micro (mi)RNA species, which, in turn, affect expression of multiple genes.

This review aims to summarize current knowledge on the multiple roles of KSRP as a regulator of gene expression, with a focus on its emerging importance on the induction and course of innate and adaptive immune responses, in addition to tumorigenesis.

## 2. KSRP Regulates Gene Expression on Various Levels

### 2.1. Gene Transcription

In 1996 KSRP was originally identified as a transcription factor of the c-myc oncogene [5]. KSRP binds to FUSE motif, and the four Y-rich regions within the C-terminus of KSRP activate c-myc transcription [8]. One year later the ability of KSRP to act as a pre-mRNA splicing regulatory protein was demonstrated. As a component of a multiprotein complex, KSRP was found to bind to an intronic splicing enhancer element of the proto-oncogene c-src and to regulate the alternative splicing process of c-scr pre-mRNA [13].

### 2.2. mRNA Level

KSRP plays an important role in different steps of post-transcriptional control of gene expression, including the regulation of mRNA stability and translatability. In addition to modulation of post-transcriptional target gene expression, KSRP also promotes the biogenesis of distinct micro (mi)RNA species, which, in turn, may affect expression of numerous genes as outlined in the following.

#### 2.2.1. mRNA Stability

Many mRNAs of pro-inflammatory mediators that possess AU-rich elements (AREs) in the 3′UTR are targets of KSRP-mediated mRNA decay and are, therefore, often inherently unstable [14]. For example, it has been demonstrated that KSRP decreases stability of the mRNAs encoding tumor necrosis factor-α (TNF-α), interleukin (IL)-8, type I and III interferons (IFN) [15,16] and inducible nitric oxide synthase (iNOS) [17] by binding to ARE in their 3′-UTR. For this, KSRP recruits the exosome multiprotein complex with 3′-5′-endonucleolytic activity and other enzymes involved in mRNA decay, such as the poly (A)-specific ribonuclease (PARN) [18], the deadenylase complex consisting of poly(A) specific ribonuclease subunit 2 and 3 (PAN2/PAN3) [19], and the 5′-3′-exonuclease 1 (XRN1) [20]. Moreover, KSRP recruits mRNA decapping enzymes such as decapping mRNA 2, which activates deadenylation of poly-A-tail of mRNA, and the decapping complex consisting of decapping mRNA 1 and 2 (DCP1/DCP2) [21,22]. KH domains 3 and 4 are necessary for KSRP-mediated mRNA decay, by binding to AREs with high-affinity and interacting with mRNA decay enzymes [9]. Both KH domains act independently of each other, resulting in a broad spectrum of target mRNAs [10]. However, KH domain 3 stabilizes the interaction of KH domain 4 to target mRNAs. Thus, altogether, KSRP seems to be a central component of the ARE-mediated mRNA decay (AMD) [15].

Moreover, in 2000, Lellek and coworkers identified KSRP as a component of the apolipoprotein B mRNA editing enzyme-complex [23]. Another study identified ~100 target mRNAs of KSRP, comprising, e.g., IL-6, IL-8 and Cyclooxygenase-2, whose expression levels were upregulated in KSRP-deficient cells [24]. However, KSRP-dependent mRNA degradation could only be detected in 10% of the ~100 target mRNAs, indicative of additional modes of KSRP-mediated gene regulation.

#### 2.2.2. Translation Efficacy

Furthermore, KSRP not only enhances mRNA degradation, but also silences mRNA translation and consequently impairs expression of, e.g., proinflammatory cytokine or chemokine genes [25]. Dhamija and coworkers compared the polysome profiles of cells with siRNA-mediated KSRP deficiency and control cells. In KSRP-deficient cells there was increased IL-6 protein. KSRP was reported to interact with ARE of IL-6 mRNA and mediate its translational silencing. However, further investigations are necessary, as to date, only Dhamjia and coworkers have identified the ability of KSRP to regulate mRNA translatability.

### 2.3. miRNA Biogenesis

miRNAs are small non-protein coding RNAs, which play a critical role in post-transcriptional gene regulation as constituents of RNA-induced silencing complexes (RISC) [26]. miRNA inhibit gene expression by binding to target sequences that are located most often in the 3′-UTR of mRNAs [26]. Thereby, they initiate translational repression and/or mRNA cleavage, depending on the degree of sequence homology to the target-binding site [27]. A single miRNA can target multiple transcripts and a single gene can be under the control of multiple miRNAs.

KSRP is important for proper processing of a subset of miRNAs, especially of those that contain a GC-rich stem-loop structure in the immature precursor transcript [28]. KH domain 3 binds selectively towards G-rich sequences, and KSRP interacts with ribonucleases Drosha and Dicer in nucleus and cytoplasm, respectively (Figure 2). In the nucleus, KSRP cleaves pri-miRNA into pre-miRNA. Moreover, it promotes the transport of pre-miRNA into the cytoplasm by interacting with exportine-5. In the cytoplasm, KSRP promotes maturation of pre-miRNA into mature miRNA by binding to the terminal loop of pre-miRNA and interacting with the ribonuclease Dicer [28]. Among those miRNAs whose maturation requires KSRP are miR-155 [29], let-7a [30,31] and miR-129 [32], which exert important functions in the regulation of immune processes as outlined in Table 1. This suggests that KSRP has an important function in immune cell biology that should be evaluated in detail.

To summarize, KSRP is a versatile RNA-binding protein (RBP) which modulates gene expression at multiple levels promoted by its structural diversity. At the moment, KSRP-mediated mRNA decay and KSRP-mediated maturation of miRNAs seem to be the most important biological functions of the protein.

## 3. Regulation of KSRP Activity

### 3.1. Transcript and mRNA Level

KSRP activity is regulated at the transcriptional and post-transcriptional level. Whereas transcriptional regulation of KSRP gene expression remains largely unexplored, more information about post-transcriptional mechanisms exists. Table 2 presents a short overview about factors that regulate KSRP expression on mRNA level.

### 3.2. Protein Level

KSRP activity is also regulated by several post-translational modifications, including phosphorylation and ubiquitination, in addition to interaction with long non coding RNAs (lncRNAs).

#### 3.2.1. Phosphorylation

KSRP contains multiple phosphorylation sites (see Figure 1) that are engaged by various kinases such as p38 mitogen-activated protein kinase (MAPK), protein kinase B (PKB) and ataxia telangiectasia mutated (ATM) kinase, thereby regulating KSRP activity [14]. Here, we only present a short overview of post-translational KSRP modifications and the functional consequences (Table 3). For detailed information see [14,49].

In addition, KSRP negatively regulates the expression of prothrombin by binding to the upstream sequence element (USE) in the 3′-UTR of prothrombin mRNA [52]. Phosphorylation of KSRP via activated p38 MAPK results in dissociation of KSRP from the USE, yielding increased stabilization of prothrombin mRNA. Furthermore, the natural compound resveratrol has been demonstrated to increase KSRP activity by inhibiting threonine phosphorylation at residue 692 [53]. This, in turn, enhances the degradation of different pro-inflammatory mRNAs, which may explain some of the anti-inflammatory effects of resveratrol. Moreover, it has been described that resveratrol interferes with the transforming growth factor β (TGF-ß)-induced epithelial-to-mesenchymal transition in mammary gland cells, which depends on KSRP [54].

Phosphorylation of KSRP at its phosphorylation sites leads to reduced activity of KSRP and thus to an increased pro-inflammatory cytokine expression.

#### 3.2.2. Ubiquitination

Ubiquitination of KSRP [55,56] attenuates its activity (as outlined in Table 4).

#### 3.2.3. Long Non-Coding RNAs

Long non-coding RNAs (lncRNA) are defined as ncRNAs longer than 200 nucleotides and are expressed in multiple cell types and tissues [61]. Some reports describe interaction of lncRNAs with KSRP that modulates KSRP-mediated mRNA decay (Table 5).

Altogether, KSRP activity is regulated at various levels by transcriptional and post-transcriptional mechanisms, e.g., by interaction with miRNA and other RNA-BP, in addition to post-translational mechanisms, including phosphorylation, ubiquitination and interaction with lncRNAs, in both nucleus and cytoplasm.

## 4. KSRP as a Regulator of Innate and Adaptive Immune Responses

PMN, monocytes/macrophages and dendritic cells (DC) recognize pathogens and are activated by pathogen-specific moieties [65] and soluble danger signals such as cytokines [66]. These innate immune cell types kill pathogens by various means, and in addition, serve as antigen presenting cells (APC) that present pathogen-derived antigens in the context of increased expression of costimulatory receptors and T cell-polarizing cytokines [67]. By this, T cells with an antigen-specific T cell receptor are activated and confer adaptive immune responses [68]. Due to its central role in AMD of pro-inflammatory mediators, KSRP was considered as an important negative regulator of inflammatory immune responses by limiting cytokine production of activated immune cells, since it promoted decay of the according mRNA, as observed in cell culture experiments [22] and when assaying primary cells isolated from KSRP^−/−^ mice [16,69,70,71].

### 4.1. Innate Immune Cells

KSRP confers regulation of immune responses on several levels. As outlined above, KSRP was reported to promote granulocytic and at the same time to inhibit monocytic differentiation via processing of miR-129, and indirectly via attenuation of RUNX1 [39]. The modulation of innate immune responses by KSRP is mediated in part via regulation of type I and III interferon expression in immune and non-immune cells and modulation of retinoic acid-inducible gene (RIG-)I receptor signaling [16,72,73], in addition to other cytokines generated by innate immune cells as shown for monocytes/macrophages and PMN.

We observed that in the collagen antibody-induced arthritis (CAIA) disease, mouse model KSRP knock out (KSRP^−/−^) mice developed markedly lower joint inflammation compared with wild type (WT) mice, accompanied by lower expression of pro-inflammatory cytokines. In general, KSRP^−/−^ mice were less susceptible to CAIA induction and had a much less pronounced disease severity. Myeloid cells, such as macrophages or PMN, were reduced in peripheral blood mononuclear cells isolated from KSRP^−/−^ mice, and in LPS-stimulated spleen cells isolated from KSRP^−/−^ mice. Since these cells are critically involved in CAIA induction [74], the lower number of myeloid cells in KSRP^−/−^ mice may account at least in part for this phenomenon [69]. In this regard, we also showed that the frequency of apoptotic CD11b^+^ cells was significantly enhanced in KSRP^−/−^ mice.

**Figure 3:**
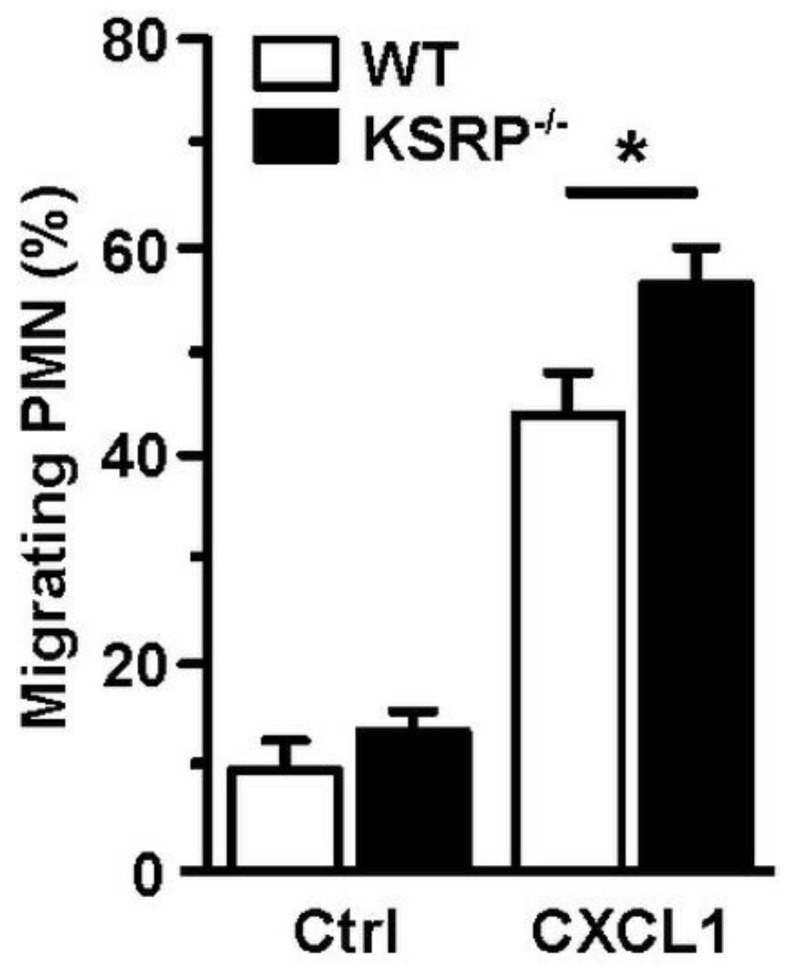
KSRP coregulates PMN migration. Bone marrow cells derived from WT and KSRP^−/−^ mice towards C-X-C motif chemokine ligand 1 (CXCL1) were assessed in a transwell (Ø 5 µm) migration assay. Bone marrow derived cells were cultivated in 24-well plates with Iscove’s Modified Dulbecco’s Medium supplemented with 5% FCS, 1% penicillin/streptavidin, 2 mM L-Glutamin and 50 µM β-Mercaptoethanol, in a final concentration of 5 × 10^5^ cells/mL. Seeded cells were stimulated with 250 ng/mL CXCL1. Control (Ctrl): w/o chemokine. After incubation for 4 h at 37 °C and 10% CO_2_, the frequency of migrating Ly6G^+^ PMN was assessed by flow cytometric analysis (mean + SEM, *n* = 6; * *p* < 0.05).

Whereas DC are the most potent type of APC [75], PMN are rather specialized in direct eradication of pathogens [76], e.g., by phagocytosis, the release of reactive oxygen species (ROS) and pathogen-binding chromatin-based extracellular traps, termed NETosis [77]. Furthermore, activated PMN secrete numerous cytokines/chemokines to attract and polarize leukocytes [78]. PMN are the first immune cell population that immigrates infected/inflamed tissue [79]. We observed that KSRP deficiency improved the migration of PMN, which suggested that KSRP may attenuate PMN migration in vivo *(*Figure 3*)*.

Altogether, several lines of experimental evidence indicate that KSRP regulates/influences the differentiation and function of PMN and monocytes/macrophages.

### 4.2. Adaptive Immune Cells

Only limited knowledge exists about the importance of KSRP for cells of the adaptive immune system. B cells and T cells are termed adaptive immune cells since these are activated in an antigen-specific manner, and thereby are able to evoke pathogen-specific immune responses. B cells provide a variety of important functions to the adaptive immune system including antibody production, antigen presentation, and cytokine secretion [80].

In adaptive immune responses, the cytokine environment is important for the activation and differentiation of CD4^+^ T cells into distinct Th cell subsets (e.g., Th1, Th2, Th9 and Th17) [81]. Activated CD4^+^ T cells play an important regulatory role as they are not only required for full activation of CD8^+^ T cells [82], but also of B cells [83]. These helper functions are predominantly determined by Th-released cytokines. CD8^+^ T cells give rise to cytotoxic T lymphocytes (CTL) which directly recognize and kill infected (or malignant) cells that present the CTL-specific antigen via major histocompatibility complex I [84].

We demonstrated that knockdown of the KSRP protein enhanced the proliferation of polyclonally stimulated CD4^+^ T cells, but not of KSRP^−/−^ CD8^+^ T cells. Modulation of IL-2 expression, previously reported as a KSRP target in cultures of immortalized cell lines [22], seemed not to contribute to enhanced T cell proliferation, since we were not able to detect any difference in IL-2 production on mRNA or protein level between primary KSRP^−/−^ and WT CD4^+^ T cells. Another obvious finding was that upon polyclonal stimulation KSRP^−/−^ CD4^+^ T cells produced higher amounts of IL-4, IL-5, IL-9, IL-10 and IL-13 as compared with WT cells. This overall change in cytokine pattern indicates that KSRP serves to inhibit Th2 polarization [70].

**Figure 4:**
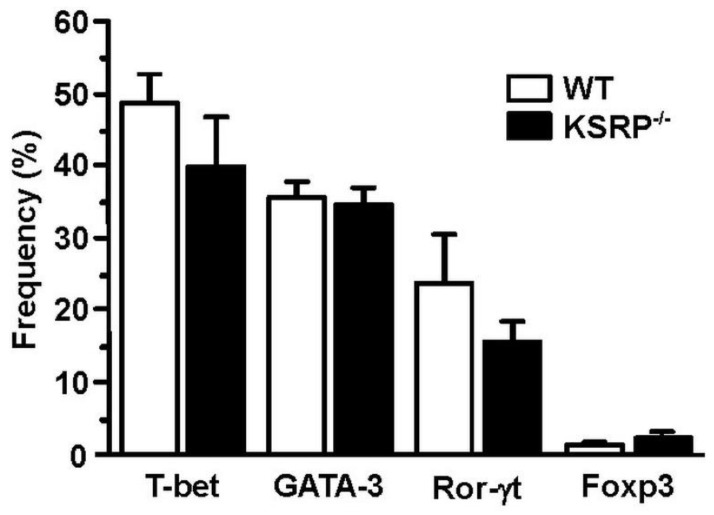
Expression of GATA-3 in stimulated CD4^+^ T cells is not affected by KSRP deficiency. Splenic CD4^+^ T cells (WT, KSRP^−/−^) were isolated by magnetic bead separation, and were polyclonally stimulated with agonistic CD3- (1 µg/mL) and CD28- (2 µg/mL) specific antibodies for 72 h. Transcription factor expression was delineated by intracellular flow cytometric analysis. Data denote the frequencies of transcription factor-positive CD4^+^ T cell (mean + SEM, *n* = 4).

In order to identify the molecular mechanisms responsible for the altered cytokine expression and proliferation of KSRP^−/−^ CD4^+^ T cells, we analyzed transcription factor expression in polyclonally stimulated CD4^+^ T cells. We observed no genotype-specific differences in Th2-associated GATA3 expression (Figure 4). This observation suggested that KSRP regulated Th polarization by targeting other mRNA species either directly or via miRNA regulation [85].

All cytokine mRNA species whose expression is differentially regulated by KSRP in CD4^+^ T cells contain ARE in their 3′-UTR, but whereas responsiveness of IL-10 and IL-13 mRNA for rapid degradation by other ARE-binding RBP such as TTP [86], AUF1 [87] and HuR [88] has been documented, much less is known about mechanisms of post-transcriptional regulation of IL-5 [89] and IL-9 [90] mRNA expression. We detected direct binding of KSRP to the IL-10 and IL-13 mRNA 3′-UTR, respectively, in pull down experiments, but not to the IL-5 and IL-9 mRNA 3′-UTR [70]. Further, we observed no direct effect of KSRP on the decay of either mRNA monitored. However, polyclonally stimulated KSRP^−/−^ CD4^+^ T cells displayed increased expression of IL-4 on mRNA and protein level as compared with the corresponding WT control, which may be explained by a longer IL-4 mRNA half-life [70]. Therefore, our data suggested that KSRP is a negative regulator of IL-4 expression. IL-4 on one hand is a master regulator of Th2 polarization [91], and on the other hand constitutes the prototypic Th2-associated cytokine [92]. Further studies are necessary to elucidate by which mechanisms KSRP modulates Th2 polarization on a molecular level.

In summary, KSRP is an important negative regulator of pro-inflammatory mediators by using its several functions, and is involved in immune response.

### 4.3. KSRP as a Negative Modulator of Immune Responses in Infection

In the case of immune responses in consequence to infections, the host innate immune system plays a significant role in the elimination of pathogen infection [93]. Danger receptors such as Toll-like receptors (TLR) play an essential role in the activation of innate immunity by recognizing specific patterns of microbial components and activating downstream intracellular signaling pathways such as nuclear factor κ-light-chain-enhancer of activated B cells (NF-κB) [94]. On one hand, the resulting expression of, e.g., pro-inflammatory cytokines needs to be fast and robust to establish a swift immune response to an invading pathogen [95]. On the other hand, cytokine expression has to be controlled strictly to avoid an excessive immune response resulting in a cytokine storm [96], extensive tissue destruction [97] or autoimmune reactions [98]. Due to its central role in AMD of mRNA species encoding pro-inflammatory mediators, KSRP is considered as an important negative regulator of inflammatory immune responses [15,22].

In this regard, KSRP was demonstrated to inhibit the activation of the retinoic acid-inducible gene (RIG-)I receptor, which is involved in antiviral defense mechanisms [73]. In the absence of KSRP RIG-I receptor induced antiviral signaling was enhanced and accordingly viral replication was reduced.

Type I interferons (IFN-α and IFN-β) play crucial roles in the innate immune response against viral infection [99,100]. Lin and colleagues detected in cells derived from KSRP^−/−^ mice that type I interferons were upregulated, which implied that KSRP plays a crucial role in maintaining low basal IFN-α/β expression levels in the absence of stimuli [72]. Additionally, type I interferon levels were increased in KSRP^−/−^ mice in response to viral infection as a result of decreased mRNA decay. Resulting from this increased expression of IFN-α and IFN-β, the KSRP^−/−^ mice were more resistant to vesicular stomatitis virus and herpes simplex virus I than WT mice.

In another study, it was demonstrated that *Helicobacter pylori* infection in mice downregulated KSRP expression and upregulated expression of inflammatory-related genes such as C-X-C motif ligand 2 and TLR2. Increasing mRNA decay of pro-inflammatory factors through KSRP overexpression in *H. pylori* mice facilitated *H. pylori* proliferation and colonization and induced aggravated gastric inflammation and mucosal damage, implying that downregulation of KSRP is necessary for an effective innate immune response against *H. pylori* [101].

Interestingly, modulation of gene expression by KSRP plays an important role not only in Gram-negative pathogens. Lipoteichoic acid (LTA) from the Gram-positive *Staphylococcus aureus* (aLTA) also constitutes a potent immunostimulation agent. KSRP was downregulated by aLTA in a monocytic cell line (THP-1) at protein level [102]. However, there were no differences of KSRP expression at gene level. Zeng and colleagues hypothesized that aLTA, similar to LPS, may regulate expression of inflammatory genes at the transcriptional level via TLR2-mediated activation of NF-κB. At the post-transcriptional level, aLTA might downregulated the destabilizing factor KSRP. Through these two regulatory mechanisms, aLTA treatment could increase and stabilize the mRNAs, and consequently elevate cytokine production. 

Investigations regarding *Salmonella enteritidis* infection in Caco-2 cells revealed also decreased KSRP expression, similar to *H. pylori* infection [103]. In addition, overexpression of KSRP in Caco-2 cells resulted in reduced levels of inflammatory factors. Interestingly, Nie and coworkers further demonstrated that the decreased expression of KSRP protein following *S. enteritidis* infection was diminished when blocking the NF-κB signaling pathway, revealing that changes in the expression of KSRP were regulated by this pathway. 

However, infection does not result, in general, in subsequent downregulation of KSRP mRNA as shown for H69 cells infected with *Cryptosporidium parvum* [46]. Instead, infection by *C. parvum* activated TLR4/NF-κB signaling and increased miR-27b-3p expression, causing a translational suppression of KSRP in infected host epithelial cells. In turn, downregulation of KSRP stabilized iNOS mRNA and promoted production of nitric oxide, exerting antimicrobial activity, by epithelial cells.

Interestingly, negative regulation of pro-inflammatory factors also plays an important role in the prevention of hepatic fibrosis. Pro-inflammatory factors such as cytokines activate hepatic stellate cells and thereby contribute to the development of hepatic fibrosis [104]. Wang and coworkers showed that soluble egg antigen stimulation and *Schistosoma japonicum* infection increased KSRP mRNA and protein levels and downregulated miR-27b-3p expression in vitro and in vivo [105]. In accordance, both knockdown of miR-27b-3p and overexpression of KSRP attenuated *S. japonicum*-induced hepatic fibrosis in vivo, due to increased mRNA decay of proinflammatory factors mediated by KSRP. 

Taken together, KSRP seems to be an important negative regulator of inflammatory immune responses in infection. While downregulation of KSRP is important for the generation of a robust immune response, against a pathogen, it is equally important to limit immune responses/production of proinflammatory factors through KSRP-mediated mRNA decay to keep the immune system homeostasis in balance.

### 4.4. KSRP as a Regulator of Auto-Inflammatory Diseases

As outlined above (see Section 4.1), we observed that, somewhat surprisingly, induction of CAIA, a well-established RA model in C57BL/6 mice, was attenuated in terms of disease onset and severity in KSRP^−/−^ mice, as compared with WT mice [69]. In addition to the reduced number of PMN in KSRP^−/−^ mice, the attenuated course of CAIA in KSRP^−/−^ mice may be explained also by the intrinsic property of KSRP^−/−^ CD4^+^ T cells to express preferentially Th2-related cytokines such as IL-4, IL-5, IL-10, and IL-13, which have been described to be important for the resolution of RA-associated inflammation in diseased joints [69,70,106].

We analyzed the role of KSRP also in a murine systemic lupus erythematosus (SLE) model. To this end, we made use of MRL-Fas^lpr^ mice, which spontaneously develop a SLE-like syndrome [107], and bred those with KSRP^−/−^ mice. The derived MRL-Fas^lpr^KSRP^−/−^ mice presented with more severe symptoms of glomerulonephritis, indicative of important protective effects of KSRP in the regulation of immune homeostasis in the kidney [71]. Moreover, we detected that the knockout of KSRP might have different effects on disease progression depending on the organ manifestation. In contrast to glomerulonephritis, lymphadenopathy, a prominent disease symptom in MRL-Fas^lpr^ mice, was attenuated in Fas^lpr^KSRP^/^mice, indicating a disease driving force of KSRP. The initial screen of the new mouse strain identified cell types (CD4^+^ IFN-γ^+^ T cells, FoxP3^+^ T cells) and targets (IL-1R, CD11a) of interest for further studies.

Concerning chronic inflammatory disease, Xia and coworkers provided evidence for a new mechanism by which liver epithelial cells maintain homeostasis during inflammation [108]. Previous studies indicated that increased levels of C-X3-C motif chemokine ligand 1 (CX3CL1) in the liver are associated with severe inflammatory liver disease [109,110]. In this study, CX3CL1 mRNA stability was demonstrated as directly regulated by KSRP through its interaction with ARE within the CX3CL1 mRNA 3′-UTR [108]. Thus, upregulation of KSRP destabilized CX3CL1 mRNA in liver epithelial cells. In addition, miR-27b-3p was identified as a negative regulator of immune reaction in response to IFN-γ stimulation: IFN-γ stimulation decreased miR-27b-3p expression and increased KSRP protein contents without changing its mRNA level in vitro and in vivo. Consequently, miR-27b-3p regulated the stabilization of CX3CL1 mRNA by attenuating KSRP mRNA translation efficiency. In agreement, downregulation of miR-27b-3p following IFN-γ stimulation resulted in KSRP induction, providing negative feedback regulation of chemokine expression in liver epithelial cells in response to inflammation.

With regard to autosomal recessive diseases such as cystic fibrosis (CF), caused by massive pro-inflammatory phenotype in the lung [111], Bhattacharyya and colleagues reported that deregulation of miR-155 might be involved in CF pathophysiology [112]. They detected high levels of miR-155 in cultured CF IB3-1 and primary lung epithelial cells and demonstrated the antagonistic role of the RNA-BP KSRP und TTP in the regulation of miR-155 biogenesis in CF cells. Suppression of KSRP led to inhibition of miR-155 maturation, whereas overexpression of TTP suppressed the processing of miR-155 through the induction of miR-1 in CF lung epithelial cells [112].

To sum up, the studies implicate that not only is KSRP an important regulator of immune reactions, KSRP itself is also regulated by miRNAs. Interaction with other RBPs also becomes obvious, raising the question of whether another destabilizing RBP can take over the function of KSRP in the case of KSRP deficiency. To answer this upcoming question, further studies are necessary.

### 4.5. KSRP Affects Tumorigenesis

Besides its role in shaping immune responses in case of autoimmunity and infection, KSRP also plays a role in tumor pathogenesis. Several reports have demonstrated an association of KSRP with different types of lung cancers. In small cell lung cancer (SCLC) increased KSRP protein levels were detected in tumor tissue, and this correlated with advanced tumor stage [113]. Knockdown of KSRP inhibited SCLC cell proliferation but had no effect on cell migration or invasion. KSRP contributed indirectly to tumor progression by promoting miR-26a maturation that led to inhibition of the tumor suppressor phosphatase and tensin homolog. Bikkavilli et al. described enhanced expression of KSRP in non (N)SCLC tissue, which correlated with poor overall survival [114]. The oncogenic properties of KSRP were attributed to KSRP-mediated downregulation of the tumor suppressor Sprouty RTK signaling antagonist 4. In addition, Yan et al. described KSRP as a metastasis-associated molecule in NSCLC [115]. In that study, interaction of KSRP with heterogeneous nuclear ribonucleoprotein C was observed, which may promote tumor metastasis by activating the IFN-α-Janus kinases—signal transducer and activator of the transcription protein 1 signaling pathway. In contrast to the results of the aforementioned studies, interestingly, KSRP was also shown to act in an anti-tumorigenic manner in NSCLC. In this regard, Chien et al. detected reduced KSRP protein expression in tumor tissue and a strong correlation between KSRP expression and overall survival [116]. The authors proposed that KSRP was necessary to promote miR-23a maturation, thus leading to destabilization of early growth response 3 mRNA, resulting in inhibition of NSCLC cell mobility.

In colorectal cancer (CRC), enhanced expression of KSRP was found in tumor tissue and this was associated with a worse overall survival [117]. KSRP seemed to drive epithelial cell proliferation in primary and metastatic cells through control of cell cycle progression and promoted, e.g., angiogenesis by enhancing vascular endothelial growth factor secretion. In addition, enhanced KSRP expression in CRC cells was associated with resistance to 5-fluoruracil treatment. Mechanistically, KSRP was demonstrated to down-regulate mRNA levels of the tumor suppressor ERBB receptor feedback inhibitor 1, by enhancing the maturation of miR-501-5p [118].

KAI1 COOH-terminal interacting tetraspanin (KITENIN) contributed to tumor progression and poor clinical outcomes in various cancers including colorectal cancer, most probably by enhancing neoangiogenesis [119]. KSRP contributed to metastasis in CRC by stabilizing the functional KITENIN complex [120]. DKC1125 (disintegrator of KITENIN complex #1125) was reported to suppress KITENIN activity by direct binding to KSRP. This interaction led to destabilization of the KITENIN complex by recruiting the receptor for activated C kinase 1 and miRNA-124, thereby suppressing metastasis in CRC. Of note, KSRP could also have a protective function in CRC by destabilizing Homeobox protein C10 (HOXC10) mRNA [121]. In CRC and in CRC-initiating cells, high expression of circular HOX (cis-HOX) RNA has been detected. cis-HOX blocked KSRP-mediated HOXC10 mRNA destabilization, thus leading to activation of the tumor-promoting Wnt/b-catenin signaling pathway.

In glioblastoma multiforme (GBM) cells, KSRP was demonstrated to inhibit migration of GBM cells, and, therefore, re-sensitized them to chemotherapy [122]. High KSRP expression was detected in GBM patients who survived long after surgery, indicating a link between KSRP and a better overall survival. Moreover, one study linked KSRP to inducing apoptosis in glioma cells in a caspase-dependent manner [123].

The Follistatin-related protein 1 (FSTL1) primary mRNA transcript also encoded for miR-198, and the switch between expression of the FSTL1 protein and miR-198 is an important regulator of tumor metastasis and wound healing [124]. KSRP processed FSTL1 mRNA to generate miR-198 by binding to the FSTL1 3′-UTR [125]. In keratinocytes, TGF-β induced the expression of miR-181a, which bound to the 3′-UTR of KSRP mRNA and thereby promoted its decay. This resulted in impaired miR-198 but enhanced FSTL1 expression. In the case of temozolomide resistance in glioma, TGF-β increased FSTL1 protein expression and decreased miR198 expression without affecting miR-181a or KSRP expression [126]. In these cases, TGF-β enhanced the expression levels of lncRNAs H19 and HOXD cluster antisense RNA 2, which competitively bind to KSRP and prevent KSRP from participating in FSTL1/miR-198 switching. This also resulted in increased expression of O-6-methylguanine DNA methyltransferase, which correlated with bad prognosis and resistance to chemotherapy.

Additionally, in squamous cell carcinoma, KSRP mediated FSTL1/miR-198 processing constitutes an important factor for metastasis [125]. Downregulation of KSRP in malignant epithelial cells inhibited miR-198 processing and thus contributed to FSTL1 expression.

In cervical cancer, interaction of KSRP with lncRNA LINC01305 promoted tumor growth [127]. Inactivation of the breast cancer susceptibility gene 1 (BRCA1) plays a significant role in breast and ovarian cancers and qRT-PCR analyses indicated that KSRP was over-expressed in BRCA1 mutated tumors [128]. In different breast cancer cell lines, mutations of the tumor suppressor gene TP53 led to changes in proteasome gene expression and enhanced proteasomal activity. In this context, anti-oncogenic properties were attributed to KSRP [129]. The p53-mediated proteasomal dysfunction resulted in increased KSRP degradation, and this impaired expression of the tumor-suppressive miRNAs let-7a and miR-30c, whose maturation is dependent on KSRP as outlined above. In breast cancer cells, IL-1 β-mediated replacement of KSRP by the mRNA stabilizing factor HuR at the IL-8 3′-UTR has been described, which in light of the tumor-promoting role of IL-8 may contribute to cancer progression [130]. We have previously demonstrated concurrent binding of KSRP and HuR to the same target 3′-UTR [17]. 

In hepatocellular carcinoma (HCC) over-expression of FBPs was identified. As outlined above, KSRP, also known as FBP2, has been shown to promote c-myc transcription [5], and c-myc was demonstrated to contribute to HCC progression [131]. The peptidyl-prolyl isomerase Pin1 is over-expressed in several cancer tissues, and may promote tumorigenesis by regulating mRNA decay in cooperation with the ARE-binding proteins AUF1 and KSRP [132].

In esophageal squamous cell carcinoma (ESCC), increased KSRP expression levels were associated with worse overall survival [133]. KSRP promoted growth, migration, and invasion of ESCC cells by enhancing the maturation of cancer-associated miRNAs, such as miR-21, miR-130b, and miR-301. Accordingly, this reduced the expression of the according miRNA target mRNAs, such as bone morphogenetic protein 6, programmed cell death protein 4, and Metalloproteinase inhibitor 3, and promoted epithelial to mesenchymal transition. Additionally, in osteosarcoma cells, KSRP was significantly upregulated, and contributed to enhanced cell proliferation and migration [134]. Likewise, KSRP was associated with enhanced proliferation of melanoma cells [135]. Here, KSRP-mediated destabilization of killin mRNA, a p53-regulated DNA replication inhibitor. Further, KSRP promoted invasiveness and metastasis of pancreatic cancer cells by interaction with the small nucleolar RNAs SNORA18 and SNORA22, and thereby enhanced the number of cell protrusions [136].

Another investigation demonstrated, in mice, that lncRNA Neat1 interacted with KSRP to promote metastasis in soft tissue sarcomas [137]. It was assumed that the Neat1/KSRP complex functioned as an RNA splicing regulator to mediate tumor cell colonization of the lung. Additionally, KSRP may also be involved in papillary thyroid carcinoma (PTC) by interacting with lncRNA AB074169 (lncAB) [138]. In normal cells, lncAB blocked mRNA decay activity of KSRP, whereas in PTC tumor cells lncAB DNA was hypermethylated, resulting in enhanced mRNA degradation of p21 by KSRP, which, in turn, promoted cell proliferation.

Overall, KSRP seems to promote cell proliferation and metastasis, but results strongly depend on tumors investigated, and opposite effects have also been described. In the context of tumorigenesis, a number of different KSRP-mediated mechanisms and KSRP targets has been described. In addition to direct regulation of mRNA stability, KSRP-mediated miRNA maturation and interaction of KSRP with lncRNA on protein level have been observed in different tumor models.

## 5. Conclusions

KSRP constitutes an important regulator of both innate and adaptive immune cells in addition to tumorigenesis. To date, KSRP may be considered as an important break of immune activation by inhibiting the production of proinflammatory cytokines by the initiation of mRNA decay and the promotion of miRNA maturation. This break is released in response to infection and also due to post-transcriptional modulation of KSRP activity. Aside from the role of KSRP to regulate the overall extent of immune activation, it also serves to finetune the character of an (adaptive) immune response, as reflected, e.g., by the intrinsic Th2 bias of CD4^+^ T cells in case of KSRP deficiency. However, further in-depth analyses are required to gain full insight in KSRP targets, especially with regard to the issue of whether this RBP acts only on the level of effector cytokines, or orchestrates the shape of immune responses also, by affecting the expression of key transcription factors. Despite the emerging evidence of the regulatory importance of KSRP, its function has been addressed in few immune cell types to date. Therefore, further studies need to address the functional role of KSRP in other innate (e.g., NK cells, innate lymphoid cells) and adaptive (B cells) immune cell types under basal conditions and in response to activation in suitable disease models. With regard to the latter, it will be important to employ mice with conditional KSRP deficiency in order to address the cell type-specific role of this RBP. Finally, it will also be important to shed light on the exact role of KSRP in tumorigenesis; depending on the tumor type, KSRP may either block or promote tumor induction and progression.

Altogether, deeper understanding into the role of KSRP in the immune system and for tumor induction and progression is a necessary prerequisite for the development of drugs which, when applied by suitable nano-carriers, may allow control of KSRP in a cell type-specific manner for therapeutic purposes. According to our data, in particular the ability of KSRP to shift T helper cell polarization towards a distinct direction (enhancing KSRP activity favors Th1 response, inhibition of KSRP activity promotes Th2 response), may be an interesting tool to restore immune homeostasis in different chronic inflammatory diseases.

## Figures and Tables

**Table 1:** Overview of immune cell functions mediated by miRNAs whose maturation is promoted by KSRP.

miRNA	miRNA Function in Immune Cells	References
miR-155	Expressed in stimulated antigen presenting cells and antigen receptor stimulated lymphocytes	[33,34]
	Hematopoietic lineage differentiation	[35]
	B cell differentiation, maturation, and antibody class switching	[36]
	Treg development	[37]
	Th cell polarization	[34,38]
miR-129	Inhibits runt-related transcription factor (RUNX)1, which determines myeloid differentiation of polymorphonuclear neutrophilic granulocytes (PMN) and monocytes	[39]
	M1-polarization of macrophages	[40]
	CD4^+^ T cell proliferation	[41]
let-7a	Promotes IL-6 expression by macrophages	[42]
	Under hypoxic conditions, imprints a M2-like immunophenotype in macrophages	[43]

**Table 2:** Summary of factors that regulate KSRP expression on the post-transcriptional level.

Regulator	KSRP Expression	References
miRNA-206	downregulated	[44]
miRNA-27b-3p	downregulated	[45,46]
HuR	upregulated	[47]
survival motor neuron proteins	upregulated	[48]

**Table 3:** Modification of KSRP by phosphorylation.

Phosphorylation	KSRP Activity	Cellular Consequence	References
p38MAPK Threonine 692	suppressed	Inhibition of KSRR-mediated mRNA decay activity	[24,25,50]
PKB Serine 193	suppressed	Interaction of KSRP with 14-3-3-ζ leads to inhibition of KSRR-mediated mRNA decay activity	[51]
		No exosome interaction possible	[10]
ATM	activated	Increased biogenesis of KSPR-dependent processed miRNAs	[15,49]

**Table 4:** Modification of KSRP by ubiquitination.

Ubiquitination of KSRP Mediated by:	KSRP	Cellular Consequence	References
Kelch-like protein 12	activity suppressed	Inhibition of KSRP-mediated internal ribosome entry site driven viral translation	[55,57]
Small ubiquitin-like modifier	activity suppressed	Attenuated KSRP-mediated maturation of miRNAs	[58,59]
Multi-protein E3 ubiquitin ligase complex	KSRP degradation		[60]

**Table 5:** lncRNAs interacting with KSRP.

lncRNA	KSRP Activity	Consequence to Target mRNA	References
H19	upregulated	Increase in mRNA decay	[62]
Epr	suppressed	Inhibition of mRNA decay	[63]
ALAE	suppressed	Inhibition of mRNA decay	[64]

## Abbreviations

aa	Amino acid
ALAE	Axon-enriched long intergenic noncoding RNA regulating axon elongation
AMD	ARE-mediated mRNA decay
APC	Antigen presenting cell
ARE	AU-rich element
ATM	Ataxia telangiectasia mutated
AUF1	AU-binding factor 1
BRCA1	Breast cancer susceptibility gene 1
CAIA	Collagen antibody induced-arthritis
CD	Cluster of differentiation
CF	Cystic fibrosis
Cis-HOX	circular HOX
CRC	Colorectal cancer
CTL	Cytotoxic T lymphocyte
CX3CL1	C-X3-C motif chemokine ligand 1
CXCL1	C-X-C motif chemokine Ligand 1
DC	Dendritic cell
ESCC	Esophageal squamous cell carcinoma
FBP	Far upstream element binding protein
FSTL1	Follistatin-related protein 1
FUSE	Far upstream element
GBM	Glioblastoma multiforme
HCC	Hepatocellular carcinoma
HuR	Human antigen R
HOXC10	Homeobox protein C10
IFN	Interferon
IL	Interleukin
iNOS	Inducible nitric oxide synthase
KH	K homology
KITENIN	Kai 1 COOH-terminal interacting tetraspanin
KSRP	KH-type splicing regulatory protein
lncRNA	Long non-coding RNA
LPS	lipopolysaccharide
LTA	Lipoteichoic acid
MAPK	Mitogen actiaved protein kinase
miRNA	MicroRNA
NF-κB	Nuclear factor k-light-chain-enhancer of activated B cells
NSCLC	Non-small cell lung cancer
PKB	Protein kinase B
PMN	Polymorphonuclear neutrophilic leukocytes
PTC	Papillary thyroid carcinoma
RA	Rheumatoid arthritis
RBP	RNA-binding protein
RIG-I	Retinoic acid-inducible gene I
RISC	RNA-induced silencing complex
ROS	Reactive oxygen species
RUNX1	Runt-related transcription factor 1
SCLC	Small cell lung cancer
SLE	Systemic lupus erythematosus
TGF-β	Transforming growth factor β
TTP	Tristetraprolin
TLR	Toll-like receptor
TNF-α	Tumor necrosis factor-α
Th	T helper cell
USE	Upstream sequence element
UTR	Untranslated region
WT	Wild type
